# HSP27 induced glaucomatous damage in mice of young and advanced age

**DOI:** 10.3389/fncel.2023.1257297

**Published:** 2023-09-07

**Authors:** Clivia Erb, Sabrina Reinehr, Carsten Theiss, H. Burkhard Dick, Stephanie C. Joachim

**Affiliations:** ^1^Experimental Eye Research Institute, University Eye Hospital, Ruhr-University Bochum, Bochum, Germany; ^2^Institute of Anatomy, Department of Cytology, Ruhr-University Bochum, Bochum, Germany

**Keywords:** aging, glaucoma, heat shock proteins, microglia, retinal ganglion cells

## Abstract

**Introduction:**

Age-related diseases such as glaucoma, a leading cause of blindness, are having an upward trend due to an aging society. In glaucoma, some patients display altered antibody profiles and increased antibody titers, for example against heat shock protein 27 (HSP27). An intravitreal injection of HSP27 leads to glaucoma-like damage in rats. We now aimed to investigate if aged mice are more prone to this damage than younger ones.

**Methods:**

We intravitreally injected HSP27 into young (1–2 months) and aged (7–8 months) mice to compare glaucomatous damage. Respective age-matched controls received PBS. Not injected eyes served as naive controls.

**Results:**

Optical coherence tomography 4 weeks after injection showed no changes in retinal thickness in all groups at both ages. Cell counts and RT-qPCR revealed a significant reduction in RGC numbers in HSP27 mice at both ages. Comparing aged and young HSP27 mice, no differences in *Rbpms* and *Pou4f1* (RGCs) expression was detected, while the *Tubb3* expression (neuronal cells) was significantly upregulated in aged HSP27 animals. Neither microglia/macrophages nor (resident) microglia counts revealed significant differences in HSP27 mice at both ages. Nevertheless, increased relative *Iba1* and *Tmem119* expression was detected in young and aged HSP27 mice. Aged HSP27 mice displayed a significantly lower *Iba1* expression than young ones, whereas *Cd68* levels were upregulated. A larger GFAP^+^ area and an upregulation of *GFAP* expression in HSP27 animals of both ages indicated a macrogliosis. Also, elevated *Il1b* and *Nos2* expression levels were observed in young and aged HSP27 mice. However, only *Il1b* levels were upregulated when comparing 7–8 months to 1–2 months old animals. A larger HSP25^+^ area was seen in aged HSP27 animals, while *Hspb2* expression levels were downregulated in both HSP27 groups. The aged HSP27 group displayed an upregulated *Hspb2* expression compared to young mice. Furthermore, a higher optic nerve degeneration score was noted in young and aged HSP27 groups.

**Discussion:**

These findings indicate that an intravitreal injection of HSP27 led to RGC loss accompanied by inflammation. Age-dependent effects (7–8 months vs. 1–2 months) were not very prominent. The results suggest a potential role of extracellular HSP27 in the development of glaucoma.

## 1. Introduction

Glaucoma is a neurodegenerative disease associated with a progressive loss of retinal ganglion cells (RGCs) and a degeneration of the optic nerve (Weinreb et al., 2014). If left untreated, this damage can lead to visual field loss and even blindness. Worldwide, glaucoma is considered one of the foremost causes of irreversible blindness (EGS, 2021). Major risk factors for developing glaucoma are aging and a high intraocular pressure (IOP; Schuster et al., 2020). However, it is known that not only elevated IOP is responsible for glaucomatous degeneration. The causative pathomechanisms of glaucoma are not yet fully understood (Kang and Tanna, 2021). Other pathogenic factors such as autoimmune reactions (Grus et al., 2004) or oxidative stress (Tezel et al., 2010) may contribute to disease development.

In 1998, Wax et al. described elevated autoantibody titers against heat shock protein (HSP) 60 in the serum of normal-tension glaucoma patients (Wax et al., 1998). Subsequently, several studies demonstrated increased serum autoantibodies levels in glaucoma patients compared to healthy individuals, which included antibodies against HSP60 (Wax et al., 1998; Tezel et al., 2000), HSP70 (Joachim et al., 2007), and HSP27 (Tezel et al., 2000; Grus et al., 2004). HSPs are part of the immune system and act under physiological circumstances as chaperones and have an anti-apoptotic effect. Stress can induce HSPs and they stimulate further immune defense processes to protect cells from the toxic effects of various stressors (Lindquist and Craig, 1988). Depending on their size, HSPs are divided in different subgroups and have different functions (Jee, 2016). HSP27 is one of the small HSPs (Kampinga et al., 2009). Intracellularly, HSPs are protective, anti-apoptotic, and act as chaperones. However, HSPs can also occur extracellularly. These extracellular HSPs serve as an alarming stress signal to other cells and lead to an activation of the immune system (De Maio and Vazquez, 2013). Several studies show that especially extracellular HSP27 is found in patient serum due to pathological conditions (Wax et al., 2001; Liao et al., 2009; Vendredy et al., 2020).

To further explore the effects of HSP27, antibodies against HSP27 were applied to isolated human retinae. This study demonstrated that the apoptotic mechanism triggered by HSP27 can induce cell death of neurons, especially RGCs (Tezel et al., 1998). Subsequently, the effect of a systemic immunization with HSP27 or HSP60 was investigated in a rat animal model. Here, the authors noted glaucoma-like damage after HSP immunization (Wax et al., 2008). Furthermore, the loss of RGCs after HSP27 immunization was accompanied by altered IgG antibody patterns in the serum (Joachim et al., 2009). In a later project, we investigated whether a local, intravitreal injection of HSP27 also leads to a degeneration. IOP-independent glaucoma-like damage could be detected in rats after 21 days, namely through a degeneration of RGCs and amacrine cells, as well as a deterioration of the optic nerve neurofilament. These findings suggest that extracellular HSP27 has degenerative effects (Grotegut et al., 2020). Subsequently, the signaling cascades by which these effects might be mediated were analyzed 14 days after HSP27 injection. An activation of the intrinsic and extrinsic apoptosis pathway could be observed in rats at 14 days. Further, an increase in nucleus factor-kappa-light-chain-enhancer of activated B cells (NF-κB) as well as an activation of microglia and T-cells was noted (Grotegut et al., 2021). Now, we aimed to investigate if advanced age increases the susceptibility to immunologically induced glaucoma.

As mentioned, glaucoma is an age-related, neurodegenerative disease. Inflammation is known to occur during the disease process. Aging is characterized by a progressive impairment of physiological functions and integrity of cells and tissues and as well by immunological changes, so called inflammageing (Schmauck-Medina et al., 2022). This leads to enhanced predisposition to some age-related diseases including cancer, diabetes, atherosclerosis, hypertension, cataract, as well as neurodegenerative diseases, like glaucoma (Liu, 2014; Maran et al., 2023). Because of the various processes known as aging, including oxidative stress (Zhang et al., 2015; Sacca et al., 2018; Ionescu-Tucker and Cotman, 2021), mitochondrial dysfunction (Sas et al., 2018; Amorim et al., 2022), and cell senescence (Lopez-Otin et al., 2013; Calcinotto et al., 2019), the cells of the eye are more susceptible to damage, which can then lead to the loss of RGCs. Additionally, microglia activate astrocytes *via* interleukin 1 beta (IL-1β), tumor necrosis factor alpha (TNFα), and the complement factor C1q during aging, which again activates the immune system, e.g., HSPs (Clarke et al., 2018).

Although glaucoma is an age-related disease, the effect of age on an HSP27 injection has never been studied. Therefore, we intravitreally injected young (1–2 months) and aged (7–8 months) mice with HSP27 to compare the expression of glaucomatous neuropathy in mice of different ages for the first time. We could note that HSP27 does trigger RGC death and degeneration of the optic nerve. In the current study we did not observe an age-dependent effect regarding retina and optic nerve damage after HSP27 injection. However, the inflammatory response was seemingly more prominent in aged mice.

## 2. Methods

### 2.1. Animals

The experiments were approved by the Animal Welfare Commission of North Rhine-Westphalia (approval code: 81.02.04.2020.A084) and complied with the Association for Research in Vision and Ophthalmology guidelines for animal experiments. Young (6–8-week-old) and aged (7–8-month-old) CD1 mice (Charles River, Sulzfeld, Germany) were used for this study. All mice were housed under environmentally controlled conditions with free access to food and water. The animals were maintained in 12-h light–dark cycles.

### 2.2. Intravitreal HSP27 injection

Mice were anesthetized with ketamine (120 mg/mL, Ratiopharm, Ulm, Germany) and xylazine (16 mg/kg, Bayer Health Care, Leverkusen, Germany). Then, a topical anesthetic was applied onto the eye (Conjuncain, 4 mg/mL Bausch&Lomb, Rochester, NY, United States) followed by a mydriatic to dilatate the pupil (Tropicamide, 5 mg/mL, Stulln, Stulln, Germany). The HSP27 protein (cat. HSP0503; Lot: 097102, AtGen, Yatap-dong, South Korea) was already dissolved in 20 mM phosphate-buffered saline (PBS, pH 7.5). One eye per animal was injected with 1 μL of 0.6 μg/μL HSP27 solution under a stereomicroscope (Zeiss, Oberkochen, Germany) with a 32-gauge needle (Hamilton, Reno, NV, United States). Control animals received the same volume of PBS (Biochrome, Berlin, Germany) since this was used as a solvent for HSP27. After the injection, an antibiotic ointment was dripped on the eye (Floxal, Bausch&Lomb). Non-injected contralateral eyes served as naive controls. After injection, the animals were monitored at close intervals to make sure that they were in good condition. Four weeks after injection, subsequent analyses were performed.

### 2.3. Optical coherence tomography measurements

Mice were anesthetized with ketamine (120 mg/kg) and xylazine (16 mg/kg). OCT images of all retinae (both ages: *n* = 5 retinae/group) were captured 4 weeks after injections using an iVivo® LAB OCT (OcuScience, Henderson, NV, United States). The middle of the retina as well as two equidistant measurements per side were measured manually using ImageJ software (NIH, Bethesda, MD, United States). Hence, five measurements were used to calculate the mean value for each retina and each ganglion cell complex. The total thickness included the retinal nerve fiber layer (RNFL), ganglion cell layer (GCL), inner plexiform layer (IPL), inner nuclear layer (INL), outer plexiform layer, and the outer nuclear layer (ONL). In addition, a separate measurement of the ganglion cell complex (RNFL, GCL, and IPL) was performed. Means were calculated per retina and used for further statistical analysis (Petrikowski et al., 2021).

### 2.4. Preparation of retina and optic nerve

After 4 weeks, eyes and optic nerves were obtained. Retinae were either frozen directly at −80°C for later RT-qPCR analysis (both ages: *n* = 8 retinae/group) or were prepared for histological cross-sections (young: *n* = 6 eyes/group; aged: *n* = 5 eyes/group). Here, eyes were fixed for 1 h in 4% paraformaldehyde (Merck, Darmstadt, Germany). Afterwards, the tissue was cryo-conserved in 30% sucrose overnight and frozen embedded in NEG-50 Tissue Tek medium (Thermo Fisher Scientific, Waltham, MA, United Sattes). Retinal cross-sections (10 μm) were mounted on glass slides (SuperfrostPlus, Thermo Fisher Scientific). Afterwards, the cuts were fixed in ice-cold acetone for 10 min and then used for immunostaining.

Optic nerves (*n* = 5 nerves/group) were fixed with 2% formaldehyde (Electron Microscopy Sciences, Hatfield, PA, United States), 2.5% glutaraldehyde (Merck), and 2 mM CaCl_2_ in 0.15 mM cacodylate buffer. Then, samples were dehydrated in an ascending ethanol series starting with 50% ethanol followed by incubation in 70% ethanol, 1% uranyl acetate (Polyscience Europe, Heidelberg, Germany), and 1% phosphotungstic acid (Merck) overnight at 4°C. The following day, dehydration was continued with an ascending ethanol series (80–100%), and the samples were then carefully transferred into epoxy resin. Therefore, the optic nerves were first incubated in propylene oxide (Merck), followed by an ascending series of propylene oxide and EPON mixtures. This embedding procedure started with propylene oxide/EPON in a 3:1 ratio, followed by a 1:1 ratio, and ended with a 1:3 ratio. Finally, the samples were permeated with pure EPON overnight at 20°C. On the third day of embedding, the EPON was renewed. We allowed the EPON-embedded samples to polymerize at 60°C for 2 days. EPON consists of glycidyl ether (Serva Electrophoresis, Heidelberg, Germany), methylnadic anhydride, 2-dodecenyl succinic anhydride, and 2,4,6-tris(dimethylaminomethyl)phenol (all: Serva Electrophoresis) in a 5.4:3.8:1.84:1 mixture. Semi-thin sections (500 nm) were cut using an Ultracut E Reichert-Jung (Leica Microsystems GmbH, Wetzlar, Germany) with a DiATOME histo diamond knife (45°, 6 mm, MX559, Diatome AG, Nidau, Switzerland).

### 2.5. Immunostaining and evaluation

Specific immunofluorescence antibodies were used to identify the different cell types of the retina (young: *n* = 6 eyes/group; aged: *n* = 5 eyes/group; Table 1). Retinal sections were blocked with a solution containing 20% donkey or goat serum and 0.1% Triton-X in PBS. Sections were incubated overnight with specific primary antibodies at room temperature. The next day, incubation was performed with the appropriate secondary antibodies for 1 h (Table 1). In all staining procedures, 4′,6-diamidino-2-phenylindole (DAPI, Serva Electrophoresis) was used for nuclear labelling. Negative controls were performed for each stain by using secondary antibodies only (Grotegut et al., 2021).

**Table 1:** Primary and secondary antibodies used for immunohistology.

Primary antibodies			Secondary antibodies		
Name and host	Company	Dilution	Name	Company	Dilution
RBPMS (rabbit)	Millipore	1:500	Donkey anti-rabbit Alexa Fluor 555	Millipore	1:500
GFAP (chicken)	Millipore	1:700	Donkey anti-chicken Alexa Fluor A488	Jackson ImmunoResearch	1:500
Iba1 (chicken)	SySy	1:500	Donkey anti-chicken Alexa Fluor Cy3	Millipore	1:400
Tmem119 (rabbit)	Abcam	1:200	Donkey anti-rabbit Alexa Fluor A488	Jackson ImmunoResearch	1:500
HSP25 (rabbit)	Enzo Life Science	1:100	Goat anti-rabbit Alexa Fluor A488	Invitrogen	1:500

For evaluation, six sections of each staining were obtained per eye with two central and two peripheral images per retina section using a fluorescence microscope (Axio Imager model M2; Zeiss). To get the same sizes for all pictures, cut-outs images were transferred to Paint Shop Pro software (version 13; Corel Corporation, Ottawa, Canada) and edited. RGCs (RBPMS^+^) were counted in the GCL. Microglia/macrophages (Iba1^+^), resident microglia (Tmem119^+^), and microglia (Tmem119^+^ and Iba1^+^) were counted in the GCL, IPL, INL and using ImageJ software (Grotegut et al., 2021). Measurements of GFAP^+^ and HSP25^+^ area were carried out using an ImageJ macro. First, images were converted into grayscale. After fixed background subtraction (Rolling Ball Radius: 50.0 pixel), the lower threshold was set at 12.78 and upper threshold at 113.96 for GFAP, for HSP25 the lower threshold was set at 10.24 and the upper threshold at 91.82. Then, the percentage of the labelled area between these thresholds was determined (Palmhof et al., 2019; Hunziker et al., 2021).

### 2.6. RNA preparation and cDNA synthesis

For RNA isolation, the retinae (both ages: *n* = 8 retinae/group) were dissected from the eyes and directly frozen at −80°C. To prepare the RNA, two retinae were pooled, so 4 samples/group/age were analyzed. Samples were transferred into lysis buffer containing 2-mercaptoethanol (Sigma-Aldrich, St. Louis, MO, United Sates) and snap frozen in liquid nitrogen. The Gene Elute Mammalian RNA Miniprep Kit (Sigma-Aldrich) was used to extract the RNA, which was then digested with RNase-free DNAse I (Sigma-Aldrich; Reinehr et al., 2018a, 2019). Using the Nanodrop ONE (Thermo Fisher Scientific), the concentration of RNA was assessed. 1 μg RNA was used for reverse transcription with a cDNA synthesis kit (Thermo Fisher Scientific).

### 2.7. Quantitative real-time PCR analysis of retinal tissue

The RT-qPCR experiments were performed in a PikoReal 96 real-time PCR system (Thermo Fisher Scientific) using SYBR Green I (Palmhof et al., 2019; Reinehr et al., 2019). The designed oligonucleotides for RT-qPCR are presented in Table 2. The genes *Actb* (β-actin) and *Ppid* (cyclophilin) were used as reference genes (Grotegut et al., 2021). Values were transferred to REST© software (Qiagen, Hilden, Germany) for further analysis.

**Table 2:** List of oligonucleotides used for mRNA expression analysis in retinae, while *Actb* and *Ppid* served as reference genes.

Gene	Forward (F) and reverse (R) oligonucleotides	GenBank acc. no.
*Actb*-F *Actb*-R	ctaaggccaaccgtgaaag accagaggcatacagggaca	NM_007393.5
*Cd68-*F *Cd68-*R	tgatcttgctaggaccgctta taacggcctttttgtgagga	NM_001291058.1
*Hspb2-*F *Hspb2-*R	catttggacacggaagtcaa ctcttcctcggggtcagg	NM_024441.3
*Iba1-*F *Iba1-*R	ggatttgcagggaggaaaa tgggatcatcgaggaattg	D86382.1
*Il1b -*F *Il1b -*R	agttgacggaccccaaaag agctggatgctctcatcagg	NM_008361.4
*Nos2-*F *Nos2-*R	ctttgccacggacgagac tcattgtactctgagggctgac	NM_010927.4
*Pou4f1-*F *Pou4f1-*R	ctccctgagcacaagtaccc ctggcgaagaggttgctc	AY706205.1
*Ppid*-F *Ppid*-R	aaggatggcaaggattgaaa ctttaagcaattctgcctgga	NM_026352
*Rbpms*-F *Rbpms*-R	cgcaaacgctacgactagag agggctactggggtaaagtg	NM_019733.3
*Tmem119-*F *Tmem119-*R	gtgtctaacaggccccagaa agccacgtggtatcaaggag	NM_146162.3
*Tubb3-*F *Tubb3-*R	gcgcatcagcgtatactacaa ttccaagtccaccagaatgg	NM_023279.3

F, forward; R, reverse; acc. no., accession number.

### 2.8. Optic nerve histology

For the analysis of the degeneration of the optic nerve tissue, cross-sections were stained with methylene blue (both ages: *n* = 5 nerves/group). Pictures of each optic nerve section were taken with a microscope (Keyence BZ-X810, Keyence, Osaka, Japan). The images of the stained optic nerve cross-sections were classified by means of an established scoring system (Kuehn et al., 2018). The images were categorized from 1 (few dark degenerations, no swollen axons, no gliosis) to 4 (dark degenerations and swollen axons throughout the whole optic nerve, gliosis) in steps of 1.

### 2.9. Statistical analysis

The immunohistological data was analyzed using Statistica (Version 13; Dell Technologies, Round Rock, TX, United States) using one-way ANOVA followed by Tukey *post-hoc* test. Data are presented as symbols for individual samples and mean ± standard error mean (SEM). Regarding RT-qPCR analyses, the relative expression values are presented as median ± quartile + minimum/maximum. The analysis of relative expression was performed by the Pair Wise Fixed Reallocation Randomization Test using REST© software (Qiagen; Pang and Clark, 2007; Grotegut et al., 2021). Optic nerve damage score was evaluated with Kruskal–Wallis test followed by Dunn’s test using Statistica software and is displayed as percentage of optic nerve damage score. *p*-values below 0.050 were considered statistically significant, with *p < 0.050, **p < 0.010, and ***p < 0.001.

## 3. Results

### 3.1. No difference in retinal thickness

**Figure 1:**
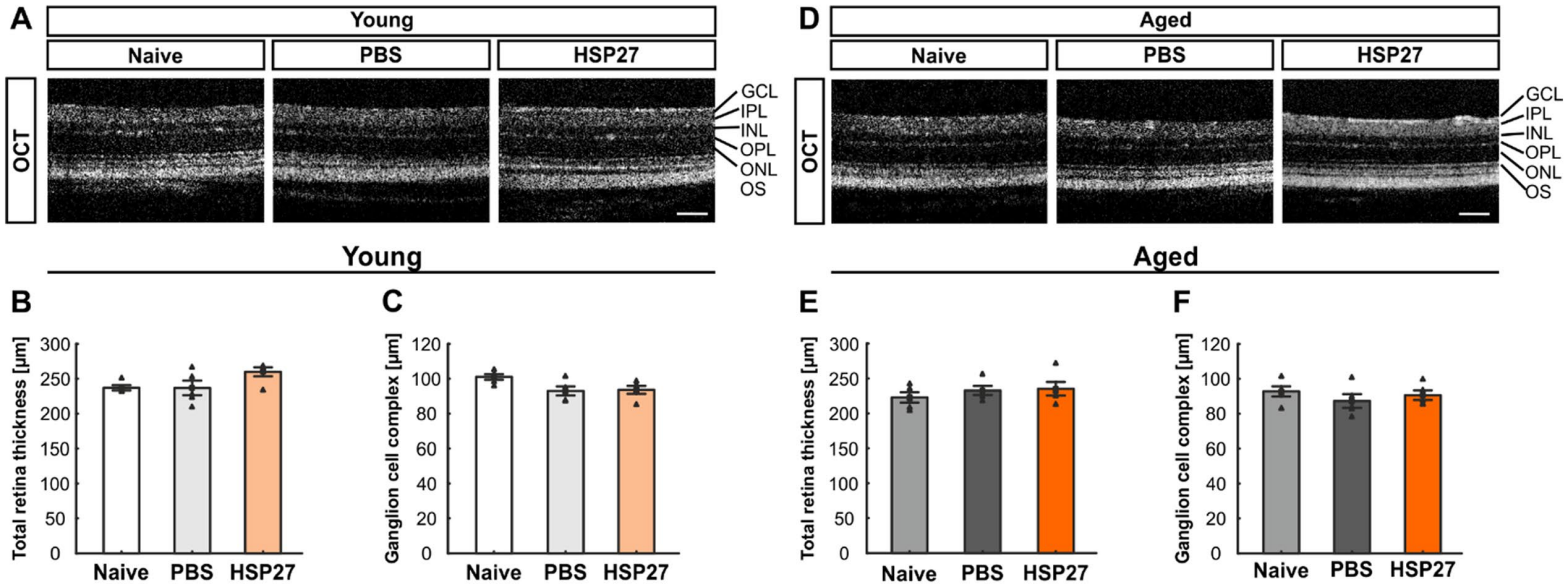
No difference in retinal thickness at both ages. **(A)** Representative OCT images in young animals of the naive, PBS, or HSP27 group are displayed. **(B)** Measurements showed no difference in total retinal thickness in young naive, PBS, and HSP27 animals. **(C)** Further, the GCC (=RNFL, GCL, and IPL) did not differ within the groups of young mice. **(D)** Exemplary OCT pictures in aged naive, PBS, or HSP27 mice. **(E)** Measurements showed no difference in total retinal thickness within all groups at the more advanced age. **(F)** In aged animals, the thickness of the GCC was not altered in HSP27 mice compared to naive and PBS animals. GCC, ganglion cell complex; GCL, ganglion cell layer; IPL, inner plexiform layer; INL, inner nuclear layer; OPL, outer plexiform layer; ONL, outer nuclear layer; OS, outer segment. Values are mean ± SEM and each symbol depicts an individual data point. *n* = 5/group. Scale bars: 200 μm.

To investigate possible structural changes in the retina, we performed OCT analyses 4 weeks after injection in both age groups. Representative OCT images in young animals of the naive, PBS, or HSP27 group were taken (Figures 1A,D). In the young animals, the measurements of the total retina thickness could not show any differences in HSP27 mice (259.97 ± 6.45 μm) in comparison to naive (237.03 ± 3.66 μm; *p* = 0.111) and PBS retinae (236.83 ± 10.38 μm, *p* = 1.000; Figure 1B).

Further, the GCC (=RNFL, GCL, and IPL) thickness did not differ between HSP27 (93.61 ± 5.11 μm) and naive animals (101.34 ± 3.58 μm; *p* = 0.083) as well as when compared to PBS mice (93.01 ± 5.84; *p* = 0.059; Figure 1C).

These measurements of the total retinal thickness as well as of the GCC were also performed in aged mice. For the total retinal thickness, the analyses showed similar results for HSP27 (235.30 ± 9.82 μm) and naive mice (222.87 ± 7.43 μm; *p* = 0.536) as well as when comparing HSP27 and PBS animals (232.83 ± 6.49 μm; *p* = 0.665; Figure 1E).

Also, no alterations in the GCC thickness were notable in these aged animals (naive = 92.79 ± 2.91 μm, PBS = 87.30 ± 3.92 μm, HSP27 = 90.59 ± 2.76 μm; all: *p* > 0.050, Figure 1F).

### 3.2. Loss of retinal ganglion cells

**Figure 2:**
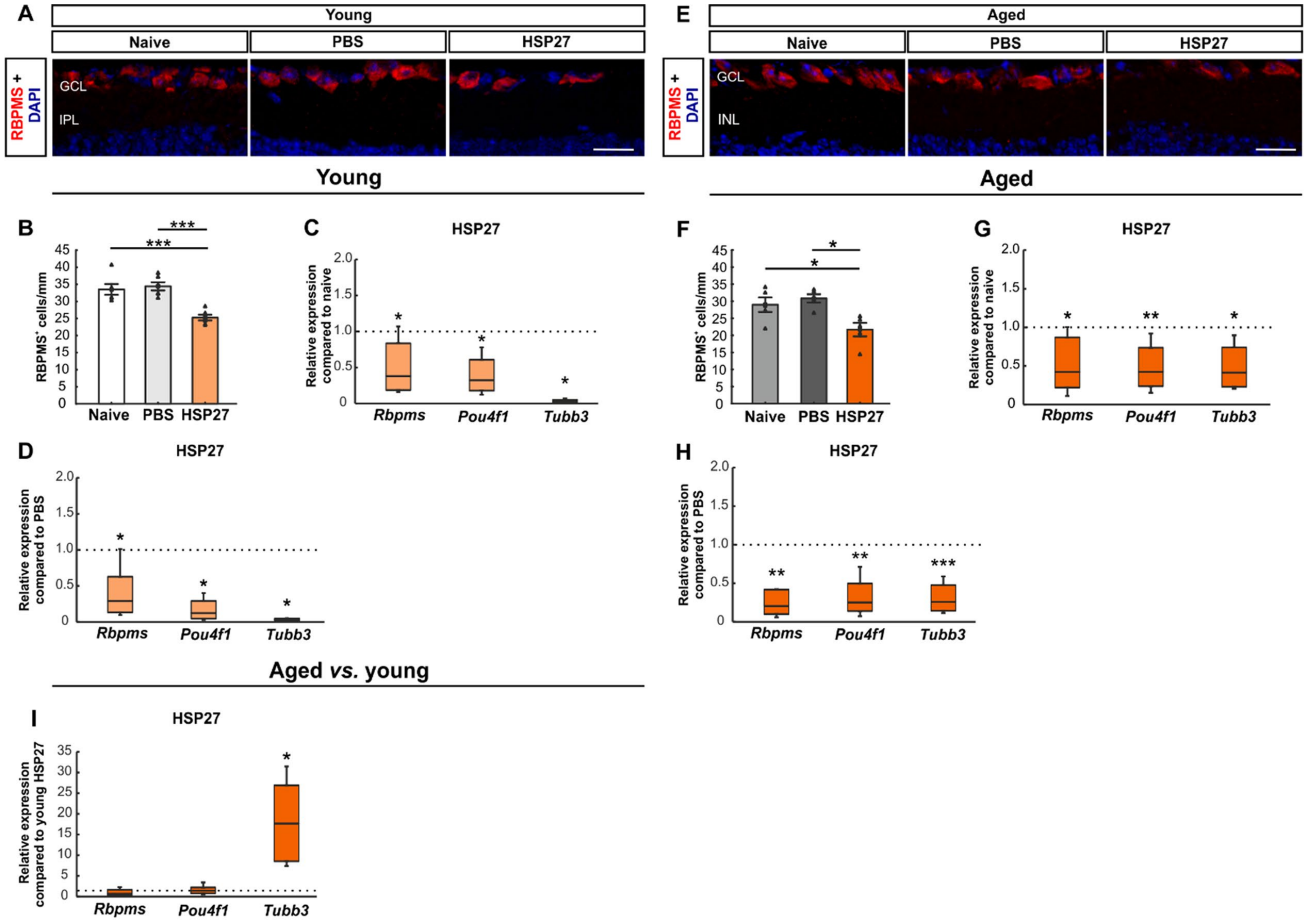
Loss of retinal ganglion cells after HSP27 injection. **(A)** RGCs in the retina of young mice were marked with RBPMS (red), DAPI (blue) was used to label cell nuclei. **(B)** RBPMS cell count revealed a significant reduction of RGC numbers in the HSP27 group compared with the naive and the PBS group (both *p* < 0.001). **(C)** A significant downregulation of *Rbpms*, *Pou4f1*, and *Tubb3* mRNA levels was noted in HSP27 group compared to naive mice (all *p* < 0.050). **(D)** Reduced *Rbpms*, *Pou4f1*, and *Tubb3* expression in HSP27 animals compared to PBS group was observed (all p < 0.050). **(E)** RGCs of aged mice were labelled with RBPMS (red), while DAPI (blue) was used to counterstain cell nuclei. **(F)** The number of RGCs was significantly decreased in aged HSP27 contrast to naive as well as to PBS mice (both *p* < 0.050). **(G)** HSP27 mice revealed a significant downregulation of *Rbpms* (*p* < 0.050), *Pou4f1* (*p* < 0.010), and *Tubb3* (*p* < 0.050) compared to naive retinae. **(H)** A downregulation of *Rbpms*, *Pou4f1* (both *p* < 0.010), and *Tubb3* (*p* < 0.001) was notable in HSP27 mice compared to PBS animals. **(I)** No difference in *Rbpms* and *Pou4f1* mRNA expression levels was seen between aged and young HSP27 mice. The mRNA expression levels of *Tubb3* were significantly upregulated in the HSP27 samples of aged mice when compared to young ones (*p* < 0.050). GCL, ganglion cell layer; IPL, inner plexiform layer. Values for immunohistology are mean ± SEM and each symbol depicts an individual data point. Values for RT-PCR median ± quartile±minimum/maximum, IF: young mice *n* = 6/group, aged mice *n* = 5/group; RT-qPCR: *n* = 4/group. The dotted lines in **C**, **D**, **G**, **H**, and **I** represent the values of the respective control groups. Scale bars: 20 μm. *p < 0.050, **p < 0.010, ***p < 0.001.

We performed immunostaining with a specific antibody against RBPMS to analyze RGCs at both ages (Figures 2A,E; Rodriguez et al., 2014). Cell counts of young animals that received an intravitreal injection of HSP27 (33.52 ± 1.58 cells/mm) revealed fewer RBPMS^+^ cells compared to naive (34.42 ± 1.21 cells/mm, *p* < 0.001) and PBS mice (25.26 ± 0.84 cells/mm, *p* < 0.001, Figure 2B).

To further investigate the damage to RGCs and neuronal cells in general, we performed PCRs using *Rbpms* and Brn-3a (*Pou4f1*), as RGC markers (Huang et al., 2014; Rodriguez et al., 2014; Muzyka et al., 2018), and β-III-Tubulin *(Tubb3)* for neuronal cells (Sharma and Netland, 2007). The mRNA expression levels of *Rbpms* (0.38-fold expression; *p* = 0.049), *Pou4f1* (0.32-fold expression, *p* = 0.030), and *Tubb3* (0.02-fold expression, *p* = 0.026) were significantly lower in young HSP27 retinae than in naive retinae (Figure 2C). A significant downregulation of *Rbpms* (0.29-fold expression; *p* = 0.047), *Pou4f1* (0.12-fold expression; *p* = 0.027), and *Tubb3* (0.03-fold expression; *p* = 0.015) was also noted in HSP27 mice compared to PBS animals (Figure 2D).

In aged mice, cell counting showed significantly fewer RBPMS^+^ cells in the HSP27 group (21.70 ± 1.98 cells/mm) compared to naive (28.98 ± 2.14 cells/mm, *p* = 0.038) and PBS mice (30.84 ± 1.22 cells/mm, *p* = 0.010; Figure 2F).

Through RT-qPCR analyses, a significant downregulation of *Rbpms* (0.42-fold expression; *p* = 0.039), *Pou4f1* (0.42-fold expression; *p* = 0.008), and *Tubb3* mRNA expression levels (0.42-fold expression; *p* = 0.017) was detected when comparing aged HSP27 retinae with naive ones (Figure 2G). Moreover, a significant decrease of *Rbpms* (0.21-fold expression; *p* = 0.003), *Pou4f1* (0.25-fold expression; *p* = 0.002), and *Tubb3* mRNA levels (0.26-fold expression; *p* < 0.001) was revealed in aged HSP27 retinae compared to PBS controls (Figure 2H).

Lastly, we compared the mRNA expression levels of young and aged HSP27 mice. While no differences were observed in the *Rbpms* (0.67-fold expression; *p* = 0.468) and *Pou4f1* expression levels (1.41-fold expression; *p* = 0.347), a significant upregulation of *Tubb3* mRNA expression was noted in aged HSP27 mice compared to young ones (17.68-fold expression; *p* = 0.023; Figure 2I).

### 3.3. Increased microglia activation

**Figure 3:**
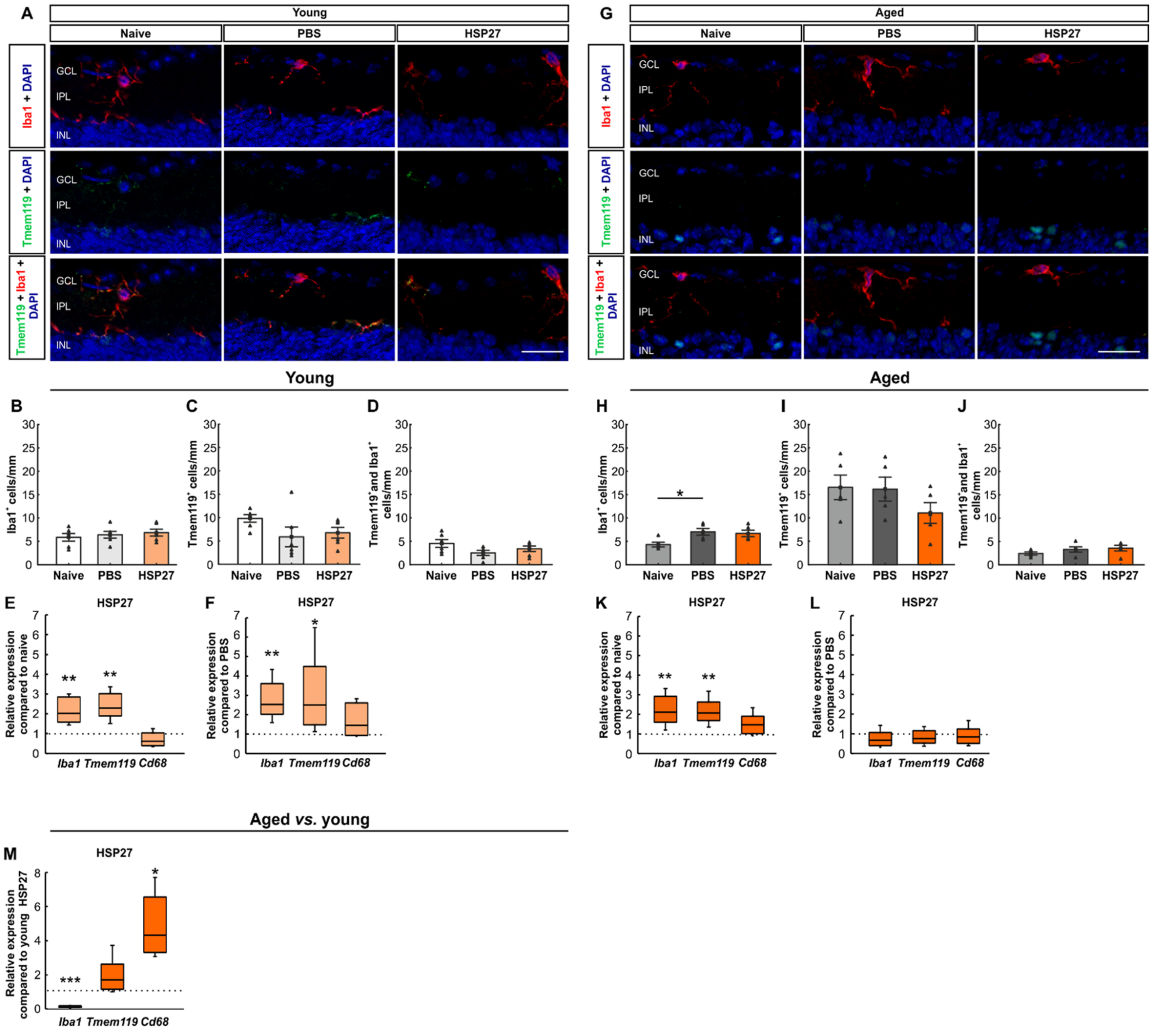
Microglial activation after HSP27 injection. **(A)** Retinae of young mice were stained with Iba1 (microglia/macrophages; red), Tmem119 (resident microglia; green) as well as a co-staining of Iba1 and Tmem119 (microglia), while cell nuclei were labelled with DAPI (blue). **(B)** The cell counts of Iba1^+^ cells were not significantly different in all groups of young animals. **(C)** No changes were noted in the number of Tmen119^+^ cells within the groups. **(D)** The number of Tmem119^+^ and Iba1^+^ cells were similar in all groups of young mice. **(E)** Elevated relative mRNA expression levels of *Iba1* (*p* < 0.010) as well as of *Tmem119* (*p* < 0.010) were noted in the HSP27 group compared to the naive group. No difference was revealed in relative expression mRNA levels of *Cd68*. **(F)** A significant upregulation of *Iba1* (*p* < 0.010) and *Tmem119* (*p* < 0.050) was observed in HSP27 group compared to PBS mice, but no alterations of *Cd68* were shown. **(G)** In aged mice, staining of Iba1 (red), Tmem119 (green), and DAPI (blue) was performed. **(H)** Iba1^+^ macroglia/macrophages numbers were increased in the PBS group compared to naive aged mice (*p* < 0.050), while no changes were seen in HSP27 mice. **(I)** No alterations were notable in the number of Tmem119^+^ cells. **(J)** The number of Tmem119^+^ and Iba1^+^ cells in aged animals was not altered between groups. **(K)** Significantly elevated relative expression levels of *Iba1* and *Tmem119* (both: *p* < 0.010) were noted in the HSP27 group compared to naive animals, while the mRNA expression of *Cd68* remained unaltered. **(L)** No differences in *Iba1*, *Tmem119*, and *Cd68* mRNA expression levels were seen in HSP27 animals compared to PBS group. **(M)** Aged HSP27 mice displayed a significantly lower relative expression of *Iba1* than young HSP27 mice (*p* < 0.001), while the mRNA expression levels of *Tmem119* was unchanged. RT-qPCR analyses revealed an upregulation of *Cd68* in aged HSP27 mice compared to young ones (*p* < 0.050). GCL, ganglion cell layer; IPL, inner plexiform layer; INL, inner nuclear layer. Values for immunohistology are mean ± SEM and each symbol depicts an individual data point. Values for RT-PCR are median ± quartile ± minimum/maximum; IF: young mice *n* = 6/group, aged mice *n* = 5/group; RT-qPCR: *n* = 4/group. The dotted lines in **E**, **F**, **K**, **L**, and **M** represent the values of the respective control groups. Scale bars: 20 μm. *p < 0.050, **p < 0.010, ***p < 0.001.

Microglia/macrophages were examined with Iba1, as it serves as a non-specific marker for these cells (Li et al., 2015; Luckoff et al., 2017). Tmem119 largely labels microglia and distinguishes microglia from resident and infiltrating macrophages. Hence, Tmem119 was used to mark resident microglia. A co-staining of Tmem119 and Iba1 was used to visualize microglia (Figures 3A,G; Bennett et al., 2016). In young animals, the number of Iba1^+^ cells did not differ significantly between groups (naive: 5.86 ± 0.81 cells/mm, PBS: 6.40 ± 0.72 cells/mm, HSP27: 6.86 ± 0.74 cells/mm; all: *p* > 0.050; Figure 3B). Also, the number of Tmem119^+^ cells was comparable in all groups (naive: 9.83 ± 0.81 cells/mm, PBS: 5.88 ± 2.11 cells/mm, HSP27: 6.76 ± 1.14 cells/mm; all: *p* > 0.050; Figure 3C). Similar results could be shown for the number of Tmem119^+^ and Iba1^+^ cells (naive: 4.53 ± 0.83 cells/mm, PBS: 2.50 ± 0.55 cells/mm, HSP27: 3.38 ± 0.59 cells/mm; all: *p* > 0.050; Figure 3D).

RT-qPCR was performed to evaluate relative mRNA expression of *Iba1*, *Tmem119*, and *Cd68* in young animals. CD68 is used as a marker that specifically binds intracellular lysosomes of macrophages (Eltony et al., 2022). Compared to naive animals, the mRNA expression levels of *Iba1* (2.03-fold expression, *p* = 0.004) and *Tmem119* (2.33-fold expression, *p* = 0.009) were significantly upregulated in HSP27 retinae, while no differences were noted in *Cd68* mRNA levels (0.60-fold expression; *p* = 0.130; Figure 3E). Similar results were obtained when comparing young HSP27 mice with respective PBS controls. The mRNA expression levels of *Iba1* (2.50-fold expression; *p* = 0.005) as well as of *Tmem119* (2.52-fold expression; *p* = 0.011) were upregulated. Again, the mRNA expression levels of *Cd68* were similar in HSP27 and PBS retinae (1.40-fold expression; *p* = 0.336; Figure 3F).

In aged mice, the number of Iba1^+^ cells was significantly higher in PBS animals (7.03 ± 0.72 cells/mm) compared to naive ones (4.30 ± 0.50 cells/mm; *p* = 0.028). A trend towards more Iba1^+^ cells was noted in HSP27 retinae (6.70 ± 0.70 cells/mm) when compared to the naive group (*p* = 0.054; Figure 3H). No differences were noted in the number of Tmem119^+^ cells within the groups of aged mice (naive: 16.53 ± 2.63 cells/mm, PBS: 16.15 ± 2.55 cells/mm, HSP27: 11.05 ± 2.23 cells/mm; all: *p* > 0.050; Figure 3I). Also, the number of Tmem119^+^ and Iba1^+^ cells did not differ between the groups at this age (naive: 2.43 ± 0.34 cells/mm, PBS: 3.29 ± 0.55 cells/mm, HSP27: 3.57 ± 0.61 cells/mm; all: *p* > 0.050; Figure 3J).

The RT-qPCR analyses in aged animals revealed a significant upregulation of *Iba1* (2.11-fold expression; *p* = 0.008) and *Tmem119* mRNA levels (2.10-fold expression; *p* = 0.004) in HSP27 mice compared to naive controls. The mRNA levels of *Cd68* did not differ between HSP27 retinae and naive ones (1.47-fold expression; *p* = 0.115; Figure 3K). When comparing HSP27 with PBS, no changes could be observed in the mRNA expression levels of *Iba1* (0.71-fold expression; *p* = 0.244), *Tmem119* (0.79-fold expression; *p* = 0.195), and *Cd68* (0.82-fold expression; *p* = 0.469; Figure 3L).

When comparing aged HSP27 mice with young HSP27 mice, a significantly lower relative expression of *Iba1* (0.21-fold expression, *p* < 0.001) was seen. A trend towards a significantly higher mRNA expression of *Tmem119* (1.64-fold expression; *p* = 0.052) was noted. A significant upregulation of *Cd68* mRNA levels could be observed in aged HSP27 retinae compared to young ones (4.34-fold expression; *p* = 0.010; Figure 3M).

### 3.4. More macroglia response

**Figure 4:**
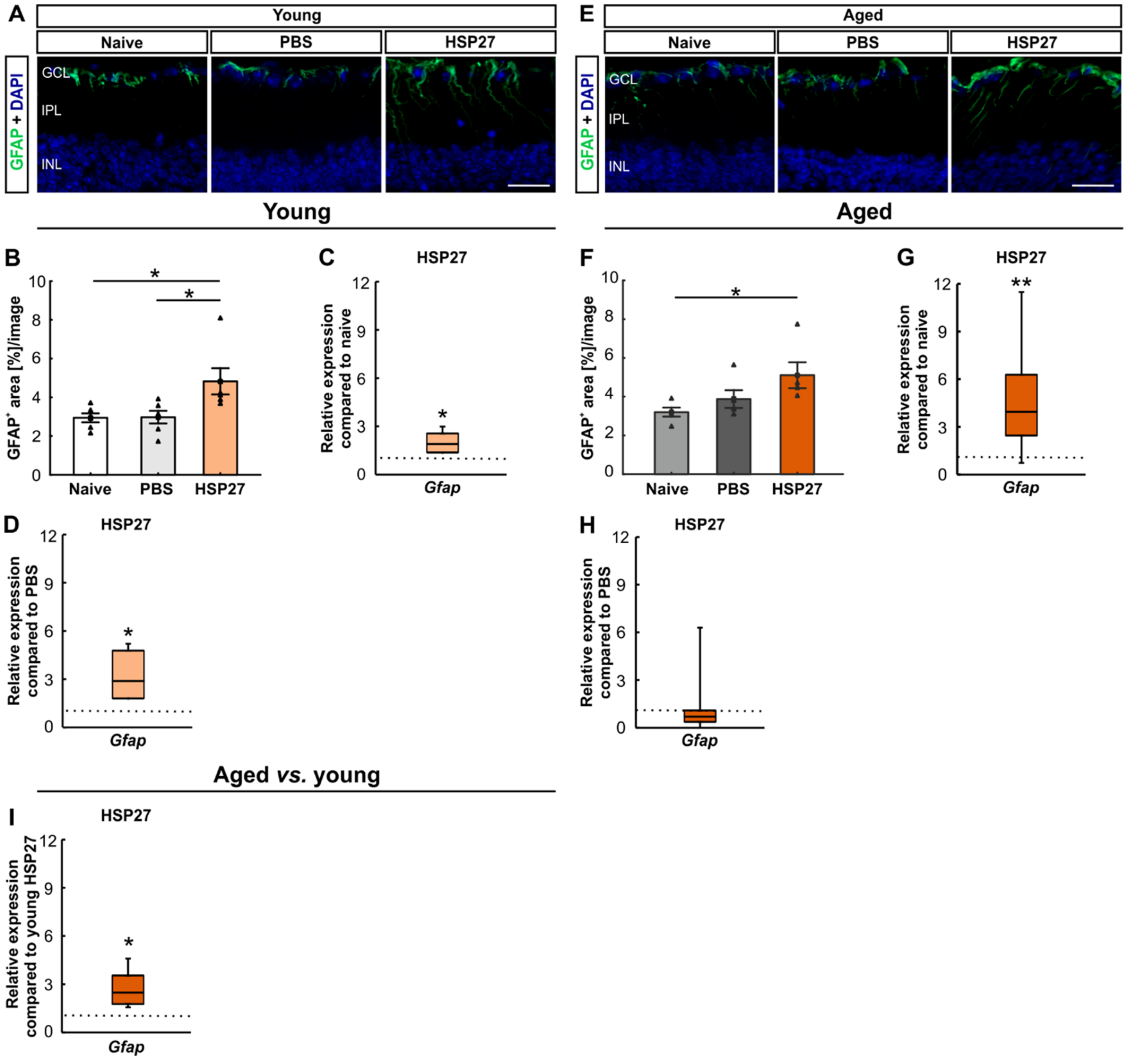
Macroglia activation after HSP27 injection. **(A)** Macroglia in the retinae of young animals were stained with GFAP (green), DAPI (blue) was used to label cell nuclei. **(B)** In young animals, a larger GFAP^+^ macroglia area was noted in HSP27 animals compared to naive and PBS ones (both *p* < 0.050). **(C)** In young HSP27 mice, an upregulation of *Gfap* was seen compared to naive samples (*p* < 0.050). **(D)** Significantly elevated *Gfap* mRNA expression levels in HSP27 retinae were detected compared to the PBS group (*p* < 0.050). **(E)** Macroglia in the retinae of aged animals were labelled with GFAP (green) and DAPI (blue) was counterstained cell nuclei. **(F)** The GFAP^+^ area in the HSP27 group was significantly larger than in naive mice (*p* < 0.050). **(G)** In aged HSP27 mice, a significant upregulation of *Gfap* mRNA levels was noted compared to naive samples (*p* < 0.010). **(H)** In aged HSP27 animals, no changes in *Gfap* expression levels were seen compared to PBS mice. **(I)** Higher *Gfap* mRNA expression levels were observed in aged HSP27 mice compared to young HSP27 ones (*p* < 0.050). GCL, ganglion cell layer; IPL, inner plexiform layer; INL, inner nuclear layer. Values for immunohistology are mean ± SEM and each symbol depicts an individual data point. Values for RT-PCR are median ± quartile ± minimum/maximum; IF: young mice *n* = 6/group, aged mice *n* = 5/group; RT-qPCR: *n* = 4/group. The dotted lines in **C**, **D**, **G**, **H**, and **I** represent the values of respective control groups. Scale bars: 20 μm. *p < 0.050, **p < 0.010.

Further, macroglia were examined by staining of retinal cross-sections with an antibody against GFAP in young and aged mice (Figures 4A,E). In young animals, the GFAP^+^ area was significantly increased in HSP27 retinae (4.82 ± 0.68%area/image) compared to naive (2.94 ± 0.23%area/image; *p* = 0.026) and PBS controls (2.97 ± 0.33%area/image; *p* = 0.029; Figure 4B).

The mRNA expression levels of *Gfap* were evaluated through RT-qPCR analyses in young retinae. A significant upregulation of *Gfap* mRNA levels was noted in HSP27 mice (1.90-fold expression, *p* = 0.013) compared to naive ones (Figure 4C). Further, a significant increase of *Gfap* mRNA expression levels was revealed in HSP27 animals (2.89-fold expression, *p* = 0.021) when compared to PBS controls (Figure 4D).

The percentage of labelled GFAP^+^ area was significantly increased in aged HSP27 mice (5.11 ± 0.67%area/image) compared to naive retinae (3.20 ± 0.23%area/image; *p* = 0.042). No alterations were observed between HSP27 and PBS animals (3.87 ± 0.46%area/image; *p* = 0.215; Figure 4F).

In aged animals, a significant upregulation of the *Gfap* mRNA expression was seen in HSP27 mice when compared to naive retinae (3.96-fold expression, *p* = 0.006, Figure 4G), but not in comparison to PBS ones (0.71-fold expression; *p* = 0.293; Figure 4H).

Furthermore, the age comparison showed an upregulation of *Gfap* in the aged HSP27 mice (0.23-fold expression, *p* = 0.015, Figure 4I).

### 3.5. Inflammatory response

**Figure 5:**
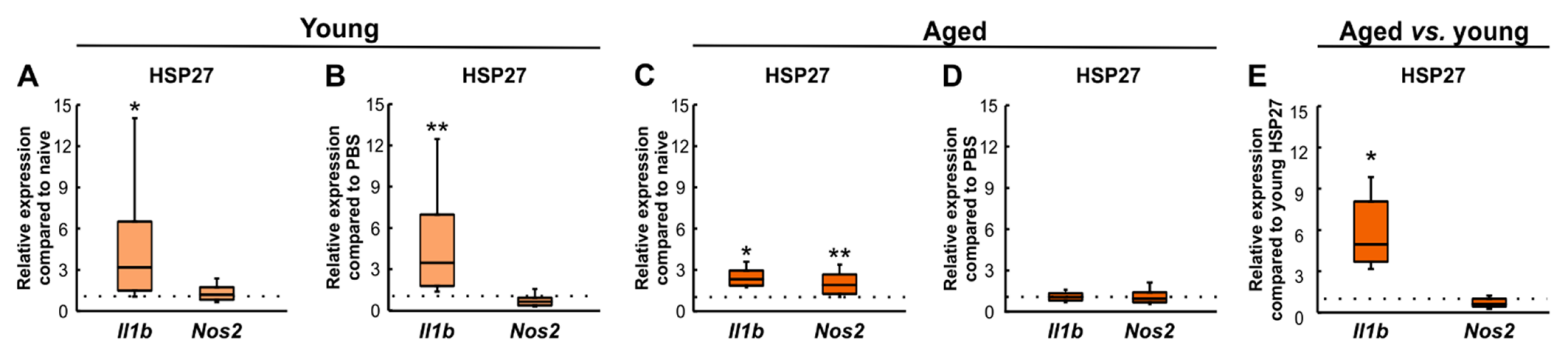
Inflammatory response after HSP27 injection. **(A)** Upregulation of *Il1b* mRNA levels (*p* < 0.050) was seen in young HSP27 mice compared to naive ones. The expression levels of *Nos2* were unaltered. **(B)** Elevated relative expression of *Il1b* (*p* < 0.010) and unaltered *Nos2* expression were shown in HSP27 retinae compared to PBS animals. **(C)** Aged HSP27 mice displayed significantly higher *Il1b* (*p* < 0.050) and *Nos2* (*p* < 0.010) mRNA expression levels than naive animals. **(D)** No alterations in *Il1b* and *Nos2* expression were seen in HSP27 animals compared to PBS group. **(E)** *Il1b* expression was elevated in aged mice compared to young ones (*p* < 0.050). *Nos2* mRNA expression levels were comparable. Values are median ± quartile ± minimum/maximum; *n* = 4/group. The dotted lines represent the values of the respective control groups. *p < 0.050, **p < 0.010.

To investigate the effects of an intravitreal injection of HSP27 on inflammation, the relative expression levels of IL-1β (*Il1b*) and iNOS (*Nos2*), which is induced in response to, e.g., pro-inflammatory cytokines in macrophages and other cell types (Eltony et al., 2022), were evaluated. Young HSP27 mice showed an upregulation of *Il1b* (3.19-fold expression, *p* = 0.038) and no changes in *Nos2* (1.20-fold expression, *p* = 0.294, Figure 5A) compared to the naive group. Compared to PBS mice, an elevated mRNA expression of *Il1b* (3.50-fold expression, *p* = 0.008) was noted. Contrary, no alterations in *Nos2* mRNA levels (0.64-fold expression, *p* = 0.138) were observed (Figure 5B).

In aged HSP27 animals, the relative mRNA expression levels of *Il1b* (2.32-fold expression, *p* = 0.024) and *Nos2* (1.95-fold expression, *p* = 0.002) were increased compared to naive mice (Figure 5C). Similar *Il1b* (1.19-fold expression, *p* = 0.262) and *Nos2* expression levels (0.98-fold expression, *p* = 0.903) were noted in HSP27 and PBS mice (Figure 5D).

In aged HSP27 mice, an upregulation of *Il1b* (4.97-fold expression, *p* = 0.029) was shown compared to young HSP27 retinae, while no differences were seen regarding *Nos2* expression (0.62-fold expression, *p* = 0.094, Figure 5E).

### 3.6. Distribution of HSP27

**Figure 6:**
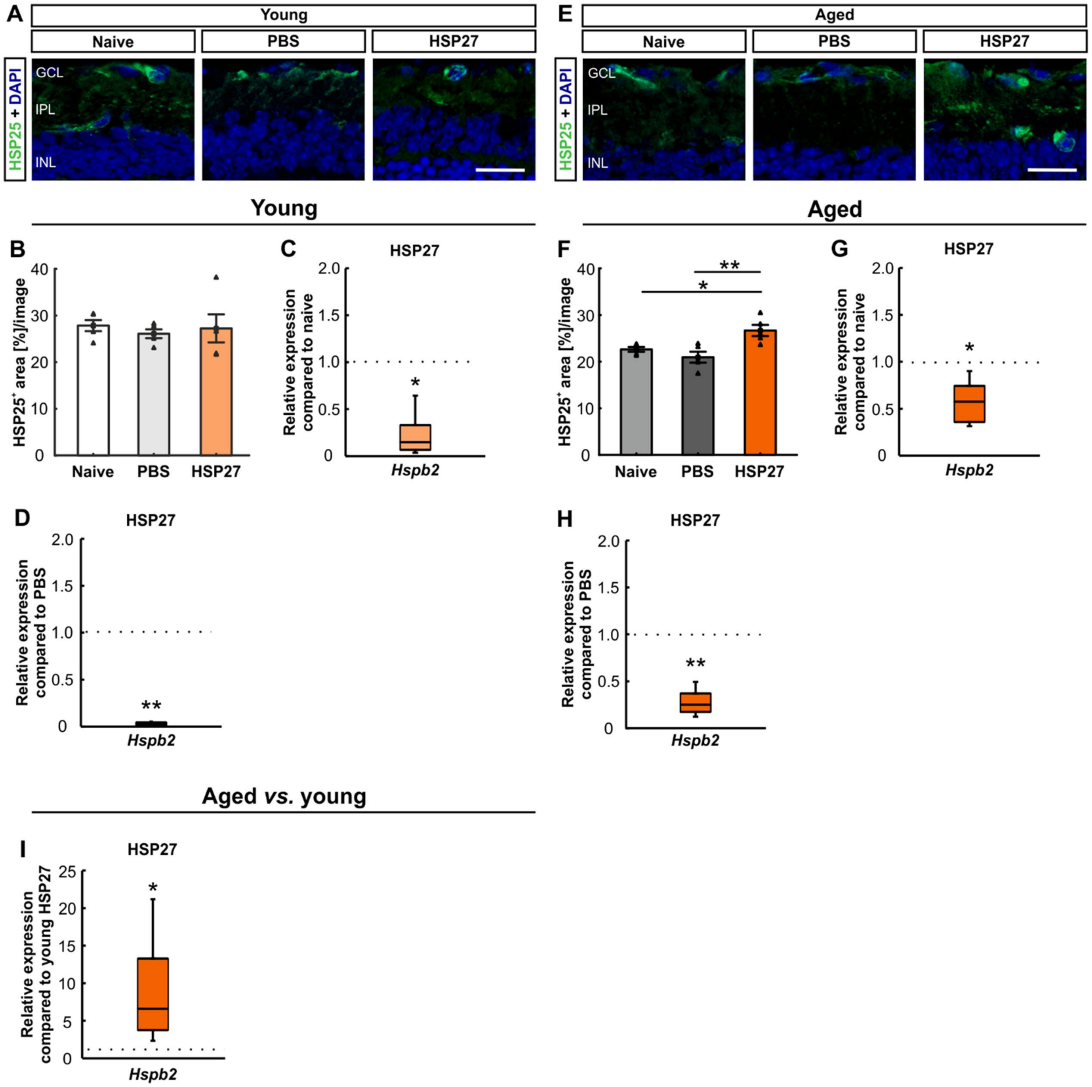
Distribution of HSP25 4 weeks after HSP27 injection. **(A)** Retinae of young mice were stained with HSP25 (green), DAPI (blue) was used to label cell nuclei. **(B)** In young animals, no difference in HSP25^+^ area was seen in all groups. **(C)** Downregulation of *Hspb2* mRNA levels was noted in young HSP27 mice compared to naive animals (*p* < 0.050). **(D)** Lower *Hspb2* mRNA expression was observed in HSP27 mice compared to PBS retinae (*p* < 0.010). **(E)** In aged mice, retinae were stained with HSP25 (green) and DAPI (cell nuclei; blue). **(F)** Aged HSP27 mice displayed a significantly larger HSP25^+^ area compared to the naive (*p* < 0.050) and the PBS group (*p* < 0.010). **(G)** Aged HSP27 mice displayed a lower *Hspb2* mRNA expression than the naive group (*p* < 0.050). **(H)** The *Hspb2* levels were downregulated in HSP27 mice compared to PBS ones (*p* < 0.010). **(I)** An upregulation of *Hspb2* in aged HSP27 mice was seen compared to the young ones (*p* < 0.050). GCL, ganglion cell layer; IPL, inner plexiform layer; INL, inner nuclear layer. Values for immunohistology are mean ± SEM and each symbol depicts an individual data point. Values for RT-PCR are median ± quartile ± minimum/maximum; IF: young mice *n* = 6/group, aged mice *n* = 5/group; RT-qPCR: *n* = 4/group. The dotted lines in **C**, **D**, **G**, **H**, and **I** represent the values of the respective control groups. Scale bars: 20 μm. *p < 0.050, **p < 0.010.

To investigate the localization of HSP27 an antibody against HSP25 was used, as HSP25 is the rodent homologue of HSP27 (Park et al., 2007; Arrigo, 2017; Zhang et al., 2022). We stained retinae of all groups at both ages with HSP25 (Figures 6A,E). In young animals, the HSP25^+^ area revealed no changes in HSP27 mice (27.23 ± 3.00%area/image) compared to the naive (27.83 ± 1.18%area/image, *p* = 0.974) and the PBS group (26.09 ± 0.93%area/image, *p* = 0.909, Figure 6B).

To evaluate the HSP27 mRNA expression, RT-qPCR analyses of *Hspb2* were performed. This revealed downregulated levels of *Hspb2* in the young mice in the HSP27 group compared to naive (0.15-fold expression, *p* = 0.030) and PBS animals (0.03-fold expression, *p* = 0.007; Figures 6C,D).

In aged mice, a larger HSP25^+^ area was seen in HSP27 retinae (26.70 ± 1.20%area/image) compared to naive (22.63 ± 0.51%area/image; *p* = 0.037) and PBS animals (20.96 ± 1.17%area/image; *p* = 0.005, Figure 6F).

Further, aged mice displayed significantly lower *Hspb2* levels in HSP27 mice compared to naive (0.58-fold expression, *p* = 0.020; Figure 6G) and PBS retinae (0.25-fold expression, *p* = 0.007; Figure 6H).

When young and aged mice were compared, significantly increased *Hspb2* mRNA levels were detected in the aged HSP27 retinae (6.62-fold expression, *p* = 0.025; Figure 6I).

### 3.7. Destructive effects on optic nerve

**Figure 7:**
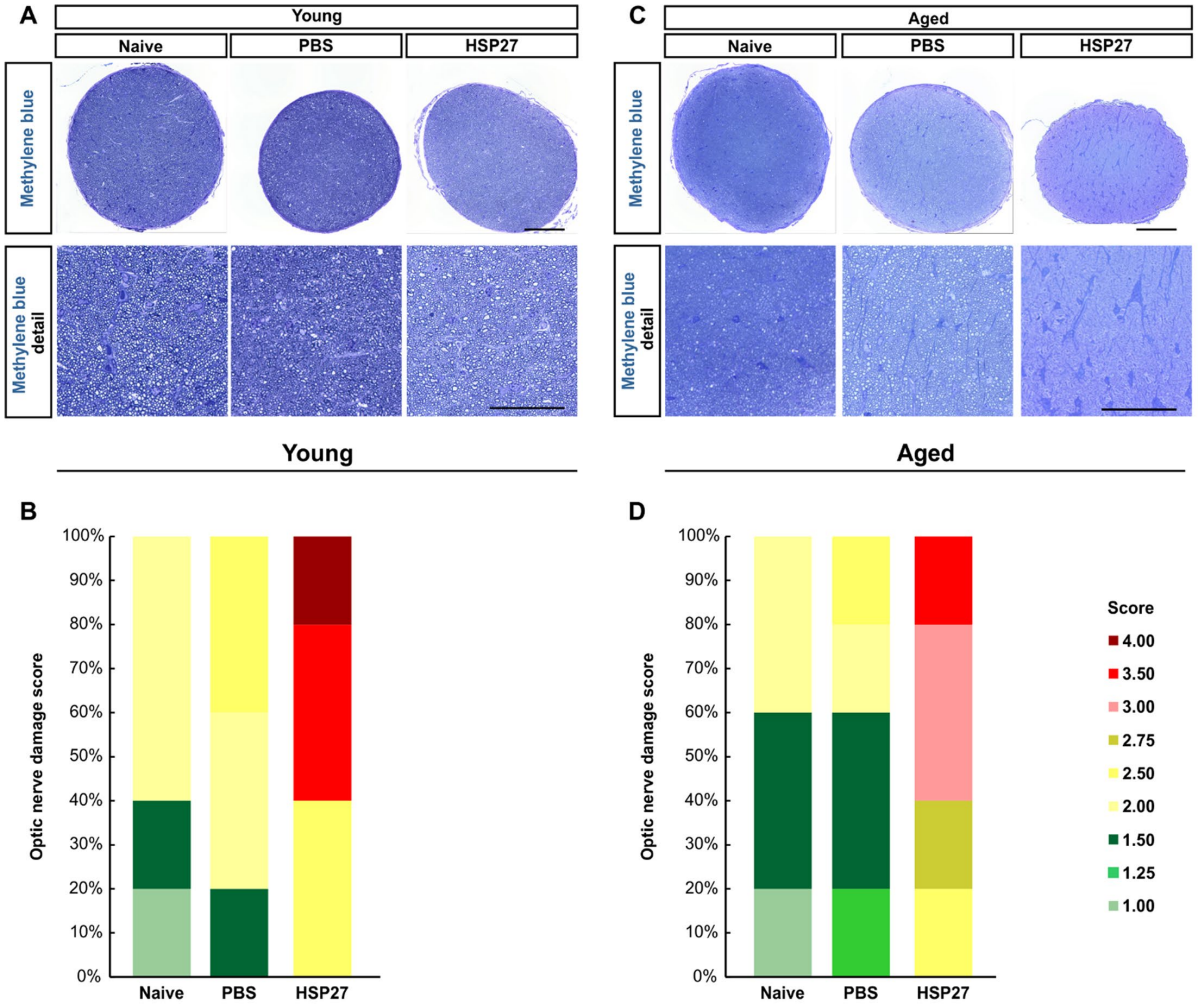
Destructive effects of HSP27 on the optic nerve. **(A)** Cross-sections of the optic nerve of young mice were stained with methylene blue. Detailed images provide a closer look into the optic nerve structure. **(B)** Optic nerve damage score in each group of young mice. HSP27 mice displayed the highest optic nerve damage. **(C)** Cross-sections of the optic nerve of aged mice were also stained with methylene blue. **(D)** Optic nerve damage score in each group of aged mice. In aged mice, the optic nerves of HSP27 animals were mostly severely damaged. Total score per group = 100%. *n* = 5/group. Scale bars: 100 μm in overview and 50 μm in detail images.

As the optic nerve is damaged while developing glaucoma, the destructive effects of HSP27 on the optic nerve were examined at both ages and PBS and HSP27 were compared to naive animals using a non-parametric test (Figures 7A,C). Therefore, cross-sections of the optic nerve were stained with methylene blue and then scored. A significantly higher median optic nerve damage score of 3.50 was found in the young HSP27 group (IQR 2.50–3.50) compared to naive (2.00, IQR 1.50–2.00, *p* = 0.001) animals, while no alterations were found in the PBS group (2.00, IQR 2.00–2.50) compared to naive ones (*p* = 0.403; Figure 7B).

In aged mice, the damage score of the optic nerves in the HSP27 group (3.00, IQR 2.75–3.00) was significantly elevated compared to naive mice (1.50, IQR 1.50–2.00; *p* < 0.001). No changes were revealed in PBS animals when compared to naive ones (*p* = 0.809, Figure 7D).

## 4. Discussion

The goal of this study was to investigate if advanced age increases the susceptibility to HSP27 induced glaucoma damage. Therefore, young (1–2 months) and aged (7–8 months) mice received an intravitreal injection of HSP27. Four weeks after injection, evaluations of RGCs, micro- and macroglia as well as inflammatory markers in the retina were performed in both age groups. In addition, an optic nerve damage score was determined. Overall, no explicit age-dependent effects were noted in our examinations. The loss of RGCs and the optic nerve damage was comparable in young and aged HSP27 mice. Age-dependent effects were noted in the response of pro-inflammatory cytokines.

Glaucoma is a progressive optic neuropathy, which can lead to blindness when untreated. It is associated with elevated IOP, which is currently the only therapy target. About 70% of glaucoma patients have primary open-angle glaucoma (POAG; Bertaud et al., 2019). 30% of those POAG patients suffer from so called normal-tension glaucoma (NTG). They display typical signs of glaucomatous damage, like RGC loss and degeneration of the optic nerve, without IOP elevation (Shen et al., 2023). The underlying pathomechanisms for both forms are not fully understood yet. Hence, other mechanisms besides IOP elevation need to be investigated. Data yielded by several research groups point towards a contribution of immunological factor, like HSPs, in glaucoma. For example, a study investigated serum samples from NTG, POAG, as well as healthy control patients and were able to show increased autoantibody titers against small HSPs, e.g., HSP27, in NTG patients compared to POAG and control subjects (Tezel et al., 1998). For further analysis, the effect of HSP27 on isolated human retinae was investigated. The results indicate that HSP27 had an apoptotic effect and induces cell death in the human retina as well as in the surrounding tissue (Tezel et al., 1998). Previous studies revealed that an intraperitoneal, intravitreal, or subcutaneous injection of HSP27 leads to glaucoma-like damage in young rats (Wax et al., 2008; Grotegut et al., 2020; Zhao et al., 2020). Furthermore, more apoptotic RGCs as well as an increase of apoptotic cells in the optic nerve were noted after HSP27 injection in these rats (Zhao et al., 2020; Grotegut et al., 2021).

Besides a high IOP, advanced age is another major risk factor for developing glaucoma (Unterlauft and Bohm, 2017; Unterlauft and EGS, 2021). Aging increases the vulnerability of neurons of the central nervous system (CNS), which include the cells of the retina. Thus, poorer regeneration has been observed in older glaucoma patients than in younger ones (Flammer and Drance, 1983). The morphology of the retina is also affected by aging processes. In animal studies, thinning of the entire retina and more irregular distribution of RGCs were observed in old rats (Mohamed et al., 2019). All these age-related changes likely contribute to an increased susceptibility to glaucoma (Xu et al., 2022). Nonetheless, experimental studies use predominantly young animals. Therefore, in the study presented here, we aimed to examine age-dependent damaging effects of HSP27 application. Hence, we compared young (1–2 months old) and aged (7–8 months old) mice that received intravitreal injections of HSP27.

The loss of RGCs is one of the hallmarks in glaucomatous degeneration (Weinreb and Khaw, 2004). In general, these cells are solely responsible for transporting visual stimuli from the retina to the brain (Yu et al., 2013; Mead and Tomarev, 2016). Hence, RGC death has detrimental effects on the normal vision. In our study, we observed a loss of RGCs *via* immunohistological staining of RBPMS as well as through RT-qPCR analyses of the RGC markers *Rbpms* and *Pou4f1*. Furthermore, also neuronal cells in general were harmed by HSP27 injections, since a downregulation of *Tubb3* mRNA levels, as a marker for neuronal cells, could also be noted at both ages. Previous results in rats showed a decrease of amacrine cells after HSP27 injection (Grotegut et al., 2020). This hints towards the possibility that not only RGCs are affected by HSP27, but also neuronal cells in general.

Moreover, we noted that HSP27 induces glaucoma-like damage due to RGC loss and optic nerve degeneration at both ages. However, the expected age dependence of the damage was not as prominent as expected. In contrary, a study by Xu et al. observed a greater RGC loss in 12- and 18-month-old mice compared to 3-month-old ones after inducing ocular hypertension (Xu et al., 2022). It is possible that with an age of 7–8 month the mice in our older cohort were already aged, but not old enough to observe age-related effects. Another explanation might be that the glaucoma-like damage induced by an intravitreal HSP27 application is not so age-dependent (compared to high IOP models). In our model, degeneration occurs without IOP elevation. After inducing chronic ocular hypertension glaucoma using the microbead model, 12-month-old CD1 mice displayed a higher RGC loss compared to 2-month-old ones (Steinhart et al., 2014). It is evidenced that the stage of the ocular connective tissue and their response to IOP play a pivotal role in the susceptibility to glaucoma damage to RGCs (Quigley and Addicks, 1981; Burgoyne et al., 2005). Thus, age alone might not play a role in the degeneration of all glaucoma animal models.

Microglia are resident immune cells in the CNS, which are also found in the retina (Kettenmann et al., 2011). Thus, they are part of the local immune system and along with macroglia they are the primary defense system of the CNS and the retina (Bobermin et al., 2020). When neurons are damaged, microglia are activated and start expressing various enzymes and cytokines. Microglia respond rapidly to pathological stimuli and migrate to a site of injury within about 24 h. Once activated, microglia can clear multiple apoptotic RGCs for at least 14 days after RGC silencing (Zhao et al., 2021). In the current study, we observed a microglia/macrophage activation in young and aged HSP27 mice *via* RT-qPCR. While the number of Iba1^+^ microglia/macrophages was not upregulated in young animals, more Iba1^+^ microglia/macrophages could be counted in aged PBS mice compared to naive ones. The number of Tmem119^+^ resident microglia as well as Tmem119^+^ and Iba1^+^ cell counts for microglia were comparable between groups at both ages. A previous study showed similar results in HSP27 injected rats. There, Iba1^+^ microglia/macrophages as well as ED1^+^ and Iba1^+^ cells were not significantly upregulated 3 weeks after injection (Grotegut et al., 2020). However, in a follow-up study, we noted more Iba1^+^ cells at an earlier time after injection, namely 2 weeks. At this time point, HSP27 injected animals also displayed more ED1^+^ and Iba1^+^ cells (Grotegut et al., 2021). These results confirm that at least on protein level, microglia/macrophages respond more in the early phases of glaucoma disease (Naskar et al., 2002; Ebneter et al., 2010; Bosco et al., 2011; Noristani et al., 2016). It is postulated that Tmem119^+^ microglia are downregulated in several models of neurodegenerative diseases, including multiple sclerosis (van Wageningen et al., 2019). Another study by Satoh et al. discovered an upregulation of *TMEM119* mRNA levels in the brain tissue from patients with Alzheimer’s disease, while no changes could be noted on protein levels (Satoh et al., 2016). This is in accordance with the results of our study. Further, the difference between cell numbers and mRNA levels could be justified by various explanations. For one, the cells were counted only in the GCL, IPL, and INL, while mRNA levels were analyzed of the whole retinae. Further, post-translational and translational regulations can lead to this inconsistency. We were not able to detect any changes in the mRNA expression levels of *Cd68* in young and aged HSP27 mice compared to controls. Interestingly, *Cd68* was significantly upregulated in aged animals compared to young ones. Intriguingly, *Iba1* expression levels, on the other hand, were significantly downregulated in aged animals. Both markers can detect microglia and macrophages. A study on human microglia observed that CD68 was more prominent in amoeboid cells (Hendrickx et al., 2017). Thus, we assume more active microglia/macrophages in older mice. To examine the role of microglia/macrophages more precisely it is important to use multiple markers to identify these cells in future studies. Different kinds of microglia express diverse markers suggesting that these microglia cells have distinct functional aims (Nayak et al., 2014; Prinz et al., 2019).

Aging is often associated with inflammatory processes, which are a response to tissue damage triggered by various stressors (Singh et al., 2019). In glaucoma, inflammation and oxidative stress often occur together, as inflammation appears to amplify oxidative stress and vice versa, creating a chronic state of inflammation and oxidative stress (Adornetto et al., 2019). In a glaucoma mouse model, increased expressions of inflammatory cytokines, including interferon-γ, IL-6, IL-4, IL-10, and IL-1β were found. This increase is thought to be related to microglial activation (Fernandez-Albarral et al., 2021). In an IOP-independent autoimmune glaucoma model, early upregulation of IL-1β levels in the aqueous humor was observed (Reinehr et al., 2018b). In our study, we noted an *Il1b* mRNA overexpression in young HSP27 mice compared to both controls. Further, in aged mice, an elevated expression of *Il1b* was seen compared to naive mice. We postulate that HSP27 application could lead to an activation of microglia, as seen by elevated mRNA levels in our study, which then release pro-inflammatory cytokines such as IL-1β, which also participates in the aging process. Interestingly, although *Il1b* was upregulated in both young and aged HSP27 mice, it was more upregulated in aged mice when comparing them to young animals. A recent study using ischemia/reperfusion injury in young and old rats compared the response of IL-1β. They found an increase in IL-1β levels in the old retinae, suggesting that aging effects lead to a higher degree of neuroinflammation (Meng et al., 2022).

As described previously, iNOS is a marker of macrophages that can be used to detect activation of the immune system. It synthesizes nitric oxide, a free radical, which is released in response to NF-κB on inflammation in activated astrocytes and microglia (Yuste et al., 2015; Hvozda Arana et al., 2020; Wiemann et al., 2020). A hypertension model in rats showed increased expression of iNOS in the retinae compared with normotensive controls (Santana-Garrido et al., 2021). Increases were not unique to hypertension-glaucoma models. In an autoimmune glaucoma mouse model, an upregulation in *Nos2* was detected in both retina as well as optic nerve tissue 10 weeks after immunization (Wiemann et al., 2020). In our study, only by comparing aged HSP27 mice to naive animals, significant changes in *Nos2* expression levels were seen. These results suggest that in our study, aged mice are more prone to inflammatory processes.

Microglia have an activating effect on macroglia, which then begin to produce neurotrophic factors and regulate synaptic activity. Macroglia, in turn, provide microglia with the physical scaffolding and energy required for their activities. This co-dependence and the communication between microglia and macroglia are becoming more and more important for the understanding of the pathogenesis of glaucoma (Zhao et al., 2021; Maran et al., 2023). Macroglia naturally support axons, but after injury or disease, they become reactive (Sofroniew and Vinters, 2010) and express more GFAP (Ridet et al., 1997). This process is known as retinal gliosis. In glaucoma patients, astrocytes and Müller cells react through hypertrophy and increased GFAP expression, which suggests that retinal gliosis is an important reactive mechanism in this disease (Tezel et al., 2003). In glaucomatous degeneration, astrocyte changes likely have both positive and destructive effects on RGC survival (Johnson and Morrison, 2009). Our experiments revealed significant larger GFAP^+^ areas in both young and aged HSP27 mice. Further, the results revealed that the relative *Gfap* expression in aged HSP27 mice is significantly increased compared to young HSP27 retinae. In glaucoma, macroglia show a reactive response causing remodeling processes, which can lead to gliosis. In human glaucoma patients, gliosis has also been described (Ashimatey et al., 2018). Reactive gliosis of astrocytes is a response to primary stress stimuli on the retina. Likely, an astrogliosis occurs in the model described here, since an increased *Gfap* expression is seen in aged HSP27 mice compared to young ones. It was described that reactive gliosis is part of aging, which would explain these findings (Lopez-Teros et al., 2022).

In agreement with other studies, our results indicate that HSP27 is part of glaucoma pathology in animal studies (Wax et al., 2008; Grotegut et al., 2020; Zhao et al., 2020). HSP27 appears to be upregulated both by neurons and glial cells in models of RGC degeneration (Chidlow et al., 2014). Intravitreal HSP27 injection not only leads to cell apoptosis, but could also be engaged in the regulation of inflammatory responses (Markiewski and Lambris, 2007). In our study, increased protein levels of HSP27 could only be observed in aged animals injected with HSP27, while at both ages, the *Hspb2* mRNA expression was downregulated. HSP27 acts directly on microglia cells to increase the secretion of pro-inflammatory cytokines. Phosphorylated HSP27 is a potent anti-inflammatory regulator (Park et al., 2003). The MAP kinases required for this are located intracellularly, but intravitreal injection tends to increase the extracellular amount of HSP27 (Binder et al., 2004). Non-phosphorylated HSP27 promotes the development of inflammatory processes. In addition, extracellular HSP27 functions as a signaling molecule for some membrane receptors such as toll-like receptors (TLR). A formation of HSP and TLR activates NF-κB, which results in the release of more inflammatory cytokines (Jin et al., 2014; Grotegut et al., 2020). Treatment of macrophages with HSP27 resulted in increased expression of several genes, including the pro-inflammatory factors IL-1β and TNF-α. However, increased anti-inflammatory factors, such as IL-10 and GM-CSF, were also detected (Jin et al., 2022). This suggests that the mode of action of HSP27 may depend on its localization. The exact mechanisms how HSP27 leads to glaucoma-like damage need to be explored in the future in more detail.

The optic nerve is also severely affected in glaucoma. A stimulus, such as an IOP increase, can disrupt the balance and lead to a chronic inflammatory response (Wareham et al., 2022). Studies in donor eyes of glaucoma patients showed an elevated expression of HSP27 in the RNFL, RGCs, and optic nerve heads (Tezel et al., 2000). After intravitreal HSP27 injection, degeneration of the optic nerve neurofilament and an increased number of apoptotic cells was observed (Grotegut et al., 2020, 2021). In our study, we confirmed that after intravitreal injection with HSP27 optic nerve degeneration occurred. Again, by comparing aged versus young HSP27 mice, no significant differences were seen, which further indicates that degeneration is not age dependent.

Based on findings from previous studies, mice from both age groups were investigated 4 weeks after HSP27 injection. Due to slow progression of degenerative effects in immunologically induced glaucoma, later time points after injection should also be examined in future studies. The additive effect of HSP27 induced damage and age might be more severe at a later point in time after injection.

In the current study, we did not note huge differences between young and aged mice. Another reason could be that the aged group was 7–8 months old. From 6 months on, mice can show aging effects, but our mice did not have a very advanced age yet (Flurkey and Harrison, 2007). It would be interesting to include even older mice (12-month-old or even older) in future studies.

Nonetheless, our results from the current study indicate that young mice can be used for studying glaucomatous neurodegeneration after HSP27 injection.

## 5. Conclusion

With this study we investigated the age-dependent effects of an intravitreal HSP27 injection. We noted a loss of RGCs as well as a degeneration of the optic nerve 4 weeks after injection at both ages. Further, activation of microglia/macrophages, microglia, and macroglia was observed, which led to an activation of the immune system. Additionally, an increase of extracellular HSP27 was noted in aged mice. These findings mimic the situation in glaucoma patients. However, the age-dependent effects were quite minor and mostly noted in the response of pro-inflammatory cytokines. In future studies, a longer point in time after HSP27 injection as well as even older mice should be used to determine if glaucomatous degeneration is age-dependent in this model. Still, the results of this study indicate that HSP27 is involved in the pathogenesis of glaucoma. Especially in this model, young mice can be used to investigate the underlying pathomechanisms and to develop new therapeutic approaches for glaucoma patients in the future.

## Data availability statement

The raw data supporting the conclusions of this article will be made available by the authors, without undue reservation.

## Ethics statement

The animal study was approved by Animal Welfare Commission of North Rhine-Westphalia. The study was conducted in accordance with the local legislation and institutional requirements.

## Author contributions

SJ: Conceptualization, Funding acquisition, Investigation, Project administration, Writing – review & editing. CE: Formal analysis, Investigation, Visualization, Writing – original draft. SR: Formal analysis, Investigation, Visualization, Writing – original draft. CT: Investigation, Writing – review & editing. HD: Resources, Writing – review & editing.

## Funding

The author(s) declare financial support was received for the research, authorship, and/or publication of this article. This study was supported by FoRUM (Ruhr-University Bochum, Germany). We acknowledge support by the Open Access Publication Funds of the Ruhr-Universität Bochum.

## Conflict of interest

The authors declare that the research was conducted in the absence of any commercial or financial relationships that could be construed as a potential conflict of interest.

The author(s) declared that they were an editorial board member of Frontiers, at the time of submission. This had no impact on the peer review process and the final decision.

## Publisher’s note

All claims expressed in this article are solely those of the authors and do not necessarily represent those of their affiliated organizations, or those of the publisher, the editors and the reviewers. Any product that may be evaluated in this article, or claim that may be made by its manufacturer, is not guaranteed or endorsed by the publisher.
